# Language switching is modulated by emotion priming: evidence from behavioral and event-related potentials study

**DOI:** 10.3389/fpsyg.2024.1373636

**Published:** 2024-12-02

**Authors:** Yun Wang, Xinfang Liu, Dianzhi Liu, Chuanlin Zhu

**Affiliations:** ^1^School of English Studies, Zhejiang International Studies University, Hangzhou, Zhejiang, China; ^2^School of Foreign Languages and Literature, Suzhou University of Science and Technology, Suzhou, Jiangsu, China; ^3^School of Education, Soochow University, Suzhou, Jiangsu, China; ^4^School of Education Science, Yangzhou University, Yangzhou, China

**Keywords:** language switching, switch costs, emotion priming, inhibitory control, inhibition modulation

## Abstract

**Introduction:**

Bilinguals often switch between different languages to effectively communicate their ideas. The variation in the increase in reaction times and error rates is termed as the language switch cost. Generally, bilingual language-switching costs demonstrate asymmetry, with a greater cost associated with transitioning from the weaker L2 to the dominant L1 than in the reverse scenario. Recent studies have demonstrated that language switching can be modulated under certain conditions. However, the effect of emotion on language-switching performance is unclear. Therefore, this study aimed to investigate the impact of emotions on bilingual language switching and how this impact manifests across different time windows.

**Methods:**

This study explored the influence of emotion on language switching between Chinese (L1) and English (L2) using a dual task involving emotion priming and word-picture matching, with concurrent measurement of event-related potentials.

**Results:**

The behavioral results indicated that a happy mood improved the accuracy and efficiency of L1 switching, while a fearful mood enhanced the efficiency of L2 switching. Electrophysiological data revealed significant interactions among emotion, language, and task in the P1, N2, and N400 stages. Specifically, a happy mood was associated with an increased P1 amplitude during L1 switching, larger N2 amplitudes during L1 repetition, L1 switching, and L2 repetition, as well as greater N400 amplitudes during L1 repetition, L1 switching, and L2 repetition, along with a larger N600 during L2 repetition. Conversely, a fearful mood exhibited a significantly larger N400 during L2 switching and a larger N600 during L2 switching.

**Discussion:**

The study findings suggest that positive emotions were beneficial for L1 switching in the early stages of visual attention allocation, conflict processing, and lexical-semantic processing. In contrast, negative emotions exhibited a more significant advantage for L2 switching in lexical-semantic processing and deeper levels of semantic processing. This study provides the first electrophysiological evidence for the impact of emotion priming on language-switching performance.

## 1 Introduction

Bilinguals often switch between different languages to effectively communicate their ideas. Grainger and Beauvillain (1987) observed a decrease in both language comprehension and production speed, along with an increase in error rate, during language switching. The variation in the increase in reaction times and error rates is termed the language switch cost (Hosoda et al., 2012), representing a quantitative measure of the inhibition of cross-language interference (Costa and Santesteban, 2004; Declerck et al., 2014). According to Green’s (1998) Inhibitory Control (IC) Model, the degree of inhibition for each language can result in different patterns of language switch costs. When transitioning from a dominant L1 to a less proficient L2, individuals must engage in inhibitory processes to suppress interference from the dominant L1 while activating the weak L2. In contrast, switching back to the dominant L1 typically incurs a higher language-switching cost than transitioning to the weak L2. This is because the dominant L1 is more frequently used in daily activities, requiring increased cognitive effort to release the previously suppressed inhibition of the dominant L1 and reactivate the weak L2. In essence, the switching cost for the weak L2 is primarily associated with inhibitory recruitment, while the switching cost for the dominant L1 is predominantly linked to releasing the previously suppressed inhibition (Meuter and Allport, 1999; Liu C. et al., 2016; Wu and Struys, 2021). Consequently, bilingual language-switching costs demonstrate asymmetry, with a greater cost associated with transitioning from the weaker L2 to the dominant L1 than in the reverse scenario.

Inhibitory control is a cognitive ability that enables individuals to refrain from responding to a routine stimulus and to block out irrelevant information (Ridderinkhof and van der Molen, 1997; Aron et al., 2004). It is also a fundamental component of language switching (Costa et al., 2006; Philipp et al., 2007). Based on Green’s (1998) IC model, switch costs primarily arise from the persistent inhibition of the non-target language. This inhibition occurs in two distinct phases during language switching. Initially, the selection of the language task schema (i.e., L1 or L2) is influenced by cues or contextual demands. The concept of language task schema competition is substantiated by research on the N2 component, with studies involving tasks like go/no-go (Huster et al., 2013), Stroop (West, 2003), flanker (van Veen and Carter, 2002), and Simon tasks (Galashan et al., 2008) indicating that, during the competitive phase between different task schemas, switch trials elicit a more negative N2 component compared to repetition trials. The increased negativity observed in the N2 component during switch trials primarily reflects conflict processing, which is more pronounced in the less proficient task. This is due to the greater need for inhibitory control to overcome interference from the dominant task, thus optimizing performance in the weaker task. Consistent with this, a larger N2 amplitude is observed during switch trials, with this effect being more pronounced in second language (L2) switch trials (Liu H. H. et al., 2016; Wu and Struys, 2022). It is posited that this phenomenon arises from the increased inhibition required to suppress L1 when switching to L2, in contrast to the decreased inhibition needed to suppress L2 when switching to L1. The subsequent phase is the lexical selection stage, where, following the selection of the relevant lexical item, non-target lexical items need to be inhibited to effectively communicate the intended language within a specific context. In previous studies, the N400 reflected the process of lexical access (Lau et al., 2008) and lexical-semantic integration (Kutas and Federmeier, 2000), serving as an indicator of cognitive mismatch in both semantic and non-semantic contexts. Some studies have suggested the existence of inhibition in both the language task schema competition stage and the lexical selection processing stage. For instance, Christoffels et al. (2007) noted a pronounced N2 component and N400 component during language-switching conditions, in contrast to non-switching conditions. Moreover, research by Wang and Lin (2021) has demonstrated that encountering incongruent conflicts that demand increased cognitive exertion prompts the activation of N600 waveforms in the frontal cortex and the bilateral anterior regions. This indicates that the N600 primarily reflects higher-level semantic processing and cognitive control processes.

These pieces of literature suggest that the P1 component plays a role in early automatic stimulus processing and sensory gating, serving as an inhibitory filter to prioritize salient stimuli (Lijffijt et al., 2009). The N2 component, elicited by rare events, reflects a change-detection response sensitive to novelty and stimulus probability (Folstein and Petten, 2008; Campanella et al., 2014). The N400 component primarily engages in semantic processing (Chan et al., 2012), while the N600 component is associated with higher-level cognitive control and semantic processing (Coulson and Kutas, 2001). In the context of bilingual switching, it is plausible to propose that the P1, N2, N400, and N600 components are linked to visual attention allocation, conflict resolution between stimuli, lexical-semantic processing, and deeper levels of semantic processing. However, further research is required to verify and fully understand the relationship between language switching and these components.

Recent studies have demonstrated that inhibitory control and language switching can be modulated by inhibition-related training. To investigate this, Liu H. H. et al. (2016) experimented to measure the effect of domain-general inhibition-related training on language switching performance in low-proficiency bilinguals. The findings indicated that this training could enhance the language-switching efficiency of participants with low inhibitory control. Subsequently, Kang et al. (2017) conducted an eight-day cued picture-naming training with a group of Chinese-English bilinguals, in which they named pictures in either of the two languages depending on visual cues. Brain activation of the participants was measured pre- and post-training, showing notable improvements in switch costs and a decrease in left dorsal anterior activation. The decrease in left dorsal anterior activation was positively associated with reductions in switch costs. These findings suggest that the effects of this training can transfer to untrained stimuli, indicating that conflict monitoring processes can be adjusted through training. The research conducted by Wu et al. (2018) further confirmed this observation. They discovered that undergoing language-switching training led to a reduction in switching costs in the dominant language, emphasizing the intricate mechanisms involved in cognitive control during bilingual language switching. In a recent study by Timmer et al. (2018), a novel approach was employed to assess the impact of short-term language-switching training on performance in non-verbal task switching. The study consisted of two groups: a task-switching training group and a single-block training group. The task-switching training group underwent language-switching training, alternating between Catalan and Spanish within each training block. The single-block training group practiced naming pictures in a single language per block. Both groups performed non-linguistic tasks (color or shape judgments) and linguistic task-switching tasks (naming pictures in Catalan or Spanish) before and after the experiment. The training tasks followed pre-training assessments immediately, with post-training assessments 1 week later. The study assessed switch cost (the reaction time difference between repeat and switch trials) and mixing cost (the reaction time difference between repeat and pure block trials) to investigate the impact of short-term language switching training on executive control by comparing changes in these cost indices before and after training between the two groups. The results indicate that both groups of participants reduced non-verbal task-switching costs and mixing costs before and after training. However, the group receiving switch-task training showed a greater decrease in switch costs. This suggests that switching mechanisms are flexible and inhibitory control abilities can be actively modulated. Despite advancements in understanding inhibitory control and task switching from a training perspective, the influence of other factors, such as emotion, on language switching performance is still a topic that requires further investigation.

Recent studies have investigated the potential intersection between bilingualism and emotional processing, primarily focusing on bilinguals performing verbal tasks in their two languages. For instance, Bialystok (2017) observed that appropriate emotional stimuli can aid bilinguals in managing semantic representations across both languages. Additionally, Barker and Bialystok (2019) examined the correlation between bilingual language processing and emotion regulation and discovered that emotional stimuli automatically engage attentional resources, thus impacting bilinguals’ performance by influencing attentional control and top-down processes related to executive control. Liu et al. (2021) and Wang et al. (2023) conducted experiments to investigate the impact of emotions on switch costs during transitions between the up-down (UD) strategy (e.g., computing 40 × 60 for 31 × 67) and the down-up (DU) strategy (e.g., computing 30 × 70 for 31 × 67) in two-digit multiplication estimation tasks with varying features under different emotional priming conditions. Their results revealed positive emotions can mitigate switching costs between strategies, facilitating smoother transitions and improving strategy execution and cognitive flexibility. Language switching and the switching between math strategies share similarities. This is because both language switching and math strategy switching represent distinct forms of task switching, involving the transition between different tasks and the allocation of attention and cognitive resources. Both require flexibility in adapting and selecting rules or behaviors in different contexts. The switching cost serves as a key indicator of cognitive flexibility, with lower costs reflecting stronger switching abilities and greater cognitive flexibility. However, it remains to be seen whether the benefits of positive emotional stimuli in mathematics strategy switching can be transferred to language-switching tasks. Furthermore, research has shown that facial expressions can activate P1 components in the initial processing of emotional information (Morel et al., 2014). Negative emotions, such as fear or anger, tend to elicit larger P1 responses compared to positive or neutral emotions, indicating a bias toward processing negative emotional information and suggesting that negative information may be processed automatically (Pourtois et al., 2004). This raises the question of whether directing visual attention to faces displaying different emotions will trigger the P1 component during the early stages of language switching and affect subsequent lexical-semantic processing. However, there is currently a lack of research in this area.

This study aimed to investigate the effect of emotion on language-switching performance. We hypothesized that emotional priming could influence language-switching performance, as evidenced by both behavioral and electrophysiological measures. To explore this, we examined how emotion priming impacted behavioral and neural responses to language switching. We expected that emotion priming would affect language switching, as language task switching is analogous to mathematics task switching. Moreover, we predicted that different emotion priming would have different effects on inhibitory control and switch cost patterns across different stages of bilingual switching. The IC model posits that heightened inhibition is necessary to suppress interference from the first language in second language switch trials, leading to larger components compared to L1 switch trials. This is primarily because L2 switching involves the utilization of inhibition to prevent L1 interference, while L1 switching mainly entails the release of previously inhibited L1 (Liu H. H. et al., 2016). This study focuses on examining the impact of emotion on inhibitory control rather than on inhibitory release. Therefore, the analysis and discussion in this study centers on L2 switch trials rather than L1 switch trials. By investigating the P1, N2, N400, and N600 components in L2 switch trials under emotional priming conditions, this study aimed to explore how emotion influences inhibitory control and modulates language switching performance. Drawing on previous research suggesting that various ERP components correspond to specific cognitive functions, we posit that the presence of larger components during L2 switch trials may indicate the influence of emotion on visual attention allocation, conflict resolution, lexical-semantic processing, and deeper levels of semantic processing in language switching.

Taking all of this together, this study aimed to investigate the impact of emotions on bilingual language switching and how this impact manifests across different time windows. Based on existing literature, we hypothesized that emotional priming significantly modulates bilingual language switching (Hypothesis 1). Specifically, significant interactions between emotional type, language type, and task type on accuracy and response time would indicate a substantial regulatory effect of emotional priming on bilingual language switching. Compared to neutral emotions, positive and negative emotions were expected to induce significant variations in accuracy and reaction time during bilingual language switching, thereby forming distinct patterns from those observed under neutral emotional conditions. Additionally, we posited that the regulatory effect of emotional priming on bilingual language switching may exhibit different characteristics across various time windows (P1, N2, N400, N600) (Hypothesis 2). Significant interactions between emotional type, language type, and task type on the amplitudes at specific time windows would suggest a significant regulatory effect of emotional priming during those stages of bilingual language switching. Furthermore, significant interactions involving emotional type, language type, task type, brain regions, and hemispheres would indicate notable effects of emotional priming on bilingual language switching in terms of brain regions and hemispheric effects.

## 2 Methods

### 2.1 Participants

A total of 24 participants were initially calculated using MorePower 6.0 software (*α* = 0.05, test power = 0.8, effect size = 0.25) (Campbell and Thompson, 2012) to improve the power of the statistical test. Fifty-six college students (36 females) with an average age of 22.26 ± 1.54 years, normal/corrected-to-normal acuity, no history of brain trauma or mental illness, who were all right-handed and had not participated in similar studies before, were selected to avoid any potential invalid data or equipment issues during the experiment. All participants signed an informed consent form under the Declaration of Helsinki (1991) and were rewarded with 60 RMB for their participation. The procedures were approved by the Research Ethics Committee of Suzhou University of Science and Technology. Data from six participants were excluded: two due to low accuracy and four due to excessive EEG artifacts. Following the accuracy standard, this study followed the guidelines set forth by Wang et al. (2023), which outlined criteria for dual-task performance, specifically emphasizing task switching and emotion judgment accuracy. After excluding two participants who scored below 70% in the word-picture matching and gender judgment tasks, the final sample consisted of 50 participants.

A self-report questionnaire was utilized to collect demographic information and language backgrounds of participants. Regarding language proficiency, participants rated their English (L2) listening, speaking, reading, and writing skills in comparison to their Chinese (L1) skills on a five-point scale. A rating of 5 indicated equivalence between L2 and L1 skills, while a rating of 1 signified significantly lower L2 proficiency than L1. Paired-sample *t*-tests demonstrated statistically significant differences between L1 and L2 proficiency ratings across all language skills (*ps* < 0.001). Language switching proficiency was assessed through a question on bilingual language switching frequency, measuring the average daily number of language switches. Responses were recorded on a Likert scale ranging from 1 (0–2 switches) to 5 (over 60 daily switches) (Lukasik et al., 2018). The findings indicated that the participants were unbalanced bilinguals with low L2 proficiency (Table 1).

**Table 1:** The demographics and language backgrounds of the sample (*M* ± SD).

Self-rating	L1 (Chinese)	L2 (English)	*p*
Age of acquisition		11.02 ± 1.35	
Listening	4.55 ± 0.51	2.17 ± 0.63	<0.001
Speaking	4.52 ± 0.51	2.26 ± 0.63	<0.001
Reading	4.38 ± 0.62	2.53 ± 0.51	<0.001
Writing	4.54 ± 0.52	2.55 ± 0.54	<0.001
Language switching frequency	1.29 ± 0.67		

### 2.2 Materials

In the language-switching task, images of actions were used, while in the emotion-priming task, images of faces were employed, as explained later.

**Figure 1:**
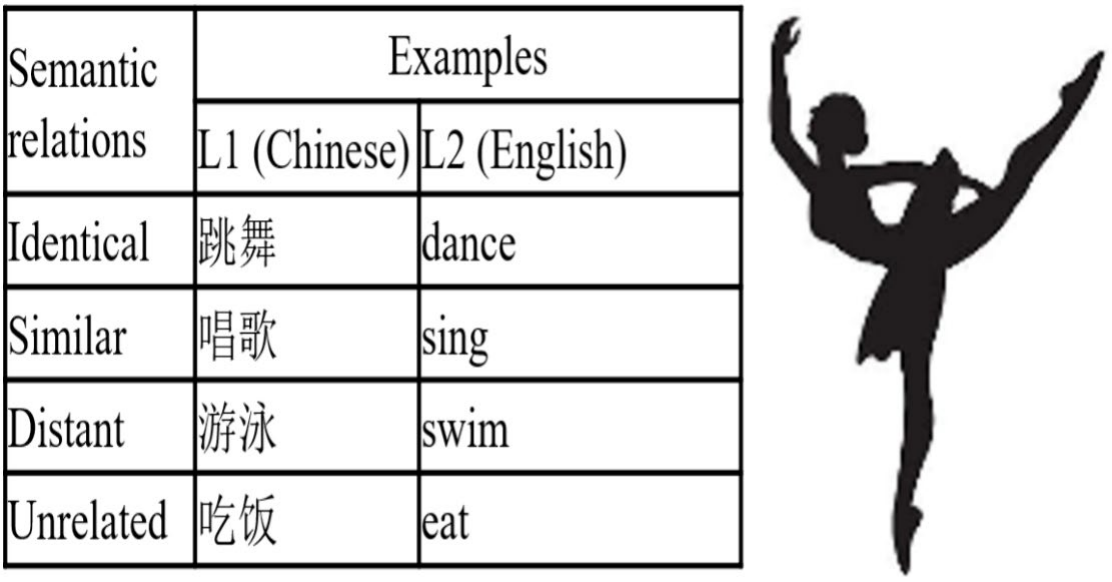
Material examples for word-picture matching.

A set of sixty 15 cm × 15 cm black-and-white line drawings was selected from the International Picture Naming Project website^1^ as action pictures standardized by Chen and Zhu (2015). The Chinese names of all the pictures were two-character words, and their English equivalents were either one- or two-syllable words with three to eight letters. To assess the familiarity of the L1 and L2 names of the pictures, another 50 students from Suzhou University of Science and Technology, with similar L2 proficiency as the participants of the experiment, rated them on a five-point scale (1 = “very unfamiliar,” 5 = “very familiar”). Paired-sample t-tests indicated that the average familiarity of the L2 names (4.78 ± 0.11) was not significantly different from that of the L1 names (4.77 ± 0.13), *t* (59) = 1.25, *p* > 0.05. Each picture was paired with a corresponding word, with four options provided based on semantic relationships: identical, similar, distant, and unrelated. The latter three served as distractors. This design allows for the investigation of the mechanisms underlying language switching between participants’ native language (L1) and second language (L2) during language comprehension (see Figure 1).

The face pictures used in the experiment were selected from the NimStim Face Stimulus Set^2^ by Tottenham et al. (2009) and consisted of 20 happy, 20 neutral, and 20 fearful pictures. Each model comprised 10 female and 10 male individuals, with a visual angle of 5.6 × 4.2 degrees. A separate group of 25 students assessed the valence and arousal of the faces on a 9-point scale. The results showed that the face pictures significantly differed in valence (*F* (2, 57) = 432.58, *p* < 0.001, *η2 p* = 0.94; positive: 6.58 ± 0.37, neutral: 4.40 ± 0.34, negative: 3.25 ± 0.39), but not in arousal (*F* (2, 57) = 158.50, *p* > 0.05, *η_p_^2^* = 0.05; positive: 5.48 ± 0.26, neutral: 5.12 ± 0.37, negative: 5.26 ± 0.49).

### 2.3 Experimental design

This study utilized a within-subject design incorporating three emotion priming conditions (positive, neutral, and negative), two languages (Chinese-L1 and English-L2), and two task types (repetition and switch). The primary focus was on the time taken by participants to complete a word-picture matching task. Language repetition refers to the consecutive use of the same language, while language switch involves switching between different languages. Language switch costs were determined by the differences in accuracy and reaction time between switch and repetition trials. A lower switch cost signifies enhanced switching ability and superior inhibitory control, while a higher cost indicates diminished switching ability and inferior inhibitory control.

Building on prior research that has investigated the relationship between emotions and task switching (Liu et al., 2021; Wang et al., 2023), we utilized a dual-task paradigm that incorporated emotion priming and language switching. It is important to highlight that effective emotion priming can only be confirmed in trials where the emotion priming task was executed accurately.

### 2.4 Procedure

**Figure 2:**
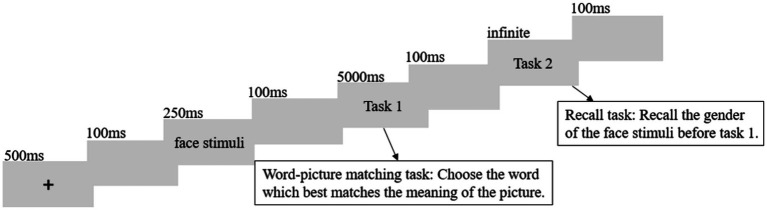
Trial structure: each trial consisted of a fixation cross, a face stimulus, and two tasks. Task 1 was available for a maximum of 5,000 ms, but would disappear when the subject pressed the button. In contrast, Task 2 remained on the screen until the subject pressed the button.

At the start of each trial, a fixation cross was presented on a 17-inch computer screen with a resolution of 1,024 × 768 pixels for 500 ms. This was followed by a blank screen for 100 ms, after which a face stimulus was presented for 250 ms. This was followed by another blank screen for 100 ms, and then an action picture with four-word choices at the bottom was presented for 5,000 ms. Participants were asked to complete the word-picture matching task and select the word that best matched the meaning of the action picture by pressing the corresponding key (“d,” “f,” “j,” “k” for the first, second, third, and fourth choices, respectively). If no response was made within 5,000 ms, the action picture and four choices would disappear. The button-right choice association was counterbalanced for each participant. Afterward, a 100 ms blank screen followed, and then a question appeared at the center of the screen asking about the gender of the previously presented face picture. Participants were required to press “j” for male and “f” for female, and the question would not disappear until a response was made. The button-gender association was counterbalanced for each participant. Subsequently, a 100 ms blank screen appeared, and the next trial began (see Figure 2).

This experiment was conducted in a sound-attenuated room and utilized E-Prime 2.0 software (Psychology Software Tools Inc., Pittsburgh, PA, USA) to record accuracy and reaction times. The experiment consisted of five blocks, each containing 120 trials, with 60 being repetition trials (30 for L1L1 and 30 for L2L2) and the other 60 being switch trials (30 for L1L2 and 30 for L2L1). The stimuli were presented in a pseudo-random order, and participants were given 24 practice trials with feedback before the formal experiment. To ensure that participants were not overly fatigued, they were given a two-minute rest period after each block.

### 2.5 Electrophysiological recordings

Electrophysiological data were recorded from 64 Ag/AgCI electrodes positioned according to the extended 10–20 system. The signal was sampled at 1 kHz and referenced online to FCz. Impedances were kept below 5 kΩ. The electroencephalographic activity was digitally filtered between 0.1 and 100 Hz, with a subsequent offline refiltering using a 30 Hz low-pass zero-phase shift filter. The ocular artifacts within the ERP EEG data were first corrected using the ICA correction method developed by Hyvärinen and Oja (2000). Any remaining artifacts were then manually removed using Analyzer 2.1 software. Continuous recordings were divided into epochs ranging from −100 to 1,000 ms relative to the onset of each trial. Baseline correction was applied using pre-stimulus activity (−100 to 0 ms) as a reference, and individual averages were re-referenced to an average of the left and right mastoid electrodes (Liu et al., 2013; Bauer et al., 2021). Signals exceeding ±75 μV in any given epoch were automatically discarded. In this study, our focus is on analyzing stimulus-locked event-related potentials (ERPs) that are time-locked to the first task of picture-naming.

### 2.6 Behavioral data analysis

#### 2.6.1 Accuracy

The first two trials of each block were excluded, and accuracy was determined as a percentage of correct responses in both Task 1 and Task 2, out of all correct responses in Task 2 (Künzell et al., 2016; Ewolds et al., 2017; Mack et al., 2023; Wang et al., 2023). To analyze accuracy, a three-way repeated-measures ANOVA was conducted with emotions (positive, neutral, negative), languages (L1, L2), and tasks (repetition, switch) as factors.

A significant three-way interaction between emotion, language, and task in a language switch task would provide direct evidence for our assumption of emotion priming in modulating inhibitory control. Furthermore, the difference between applying and releasing inhibition (i.e., L2 switch costs and L1 switch costs) can be used to evaluate the degree of modulation of emotion priming on inhibitory control, with a smaller difference implying better language switching performance.

#### 2.6.2 Reaction times

Data from the first two trials of each block and reaction times beyond *M* ± 3SD were excluded (13.31%). Only the reaction times of the correct trials in Task 1, where both task responses were correct, were analyzed. The same analysis method was used for reaction times and accuracy.

### 2.7 Event-related brain potential analysis

ERP components were defined based on the average of the left and right mastoid electrodes and were analyzed in the time windows typically used to explore the P1, N2, N400, and N600. Subsequently, repeated measures ANOVAs were performed on the mean amplitudes in the intervals of 75–105 ms (P1), 200–300 ms (N2), 300–500 ms (N400), and 600–900 ms (N600). Topographical analysis was conducted based on the mean amplitudes measured over 64 scalp electrodes. A Greenhouse–Geisser correction was applied where necessary. The regions of interest (ROIs) for P1, N2, N400, and N600 were identified after examining the current data and referring to previous studies on language switching (Swainson et al., 2001; Verhoef et al., 2009) and emotion processing (Bialystok, 2017; Barker and Bialystok, 2019). The analysis focused on P1 across three ROIs: central-parietal (CP3, CPz, CP4), parietal (P3, Pz, P4), and parieto-occipital (PO3, POz, PO4). N2, N400, and N600 were examined over three other ROIs: frontal (F3, Fz, F4), fronto-central (FC3, FCz, FC4), and central (C3, Cz, C4).

An analysis was conducted to examine the effect of positive, neutral, and negative emotion priming on the ERP components P1, N2, N400, and N600. Data from the first two trials of each block, word-picture matching errors, gender judgment errors, and trials contaminated by artifacts were excluded from the analysis (14.57% of the data). A five-way repeated-measures ANOVA was conducted on the mean amplitudes for P1, with emotions (positive, neutral, negative), languages (L1, L2), tasks (repeat, switch), hemispheres (left, midline, right), and brains (central-parietal, parietal, parieto-occipital) as factors. Similarly, for N2, N400, and N600, a five-way repeated-measures ANOVA was carried out on the mean amplitudes, with emotions (positive, neutral, negative), languages (L1, L2), tasks (repeat, switch), hemispheres (left, midline, right), and brains (frontal, fronto-central, central) as factors. If the main effects of emotion, language, and task, or any interactions containing these factors, were found to be significant (*p* < 0.05), subsequent simple effects analyses were conducted. A significant three-way interaction among emotion, language, and task would indicate that emotion plays a role in modulating language switching performance (Liu H. H. et al., 2016).

### 2.8 Correlations

In examining the connection between behavioral and neural indicators of language switching, we performed correlation analyses between the behavioral data (reaction times of L1 and L2 switch trials) and the ERP data (amplitudes of L1 and L2 switch trials at the P1, N2, N400, and N600). Our objective was to ascertain whether distinct neural encoding aspects of language switching correspond to different behavioral processing factors.

## 3 Results

### 3.1 Behavioral results

#### 3.1.1 Accuracy

**Figure 3:**
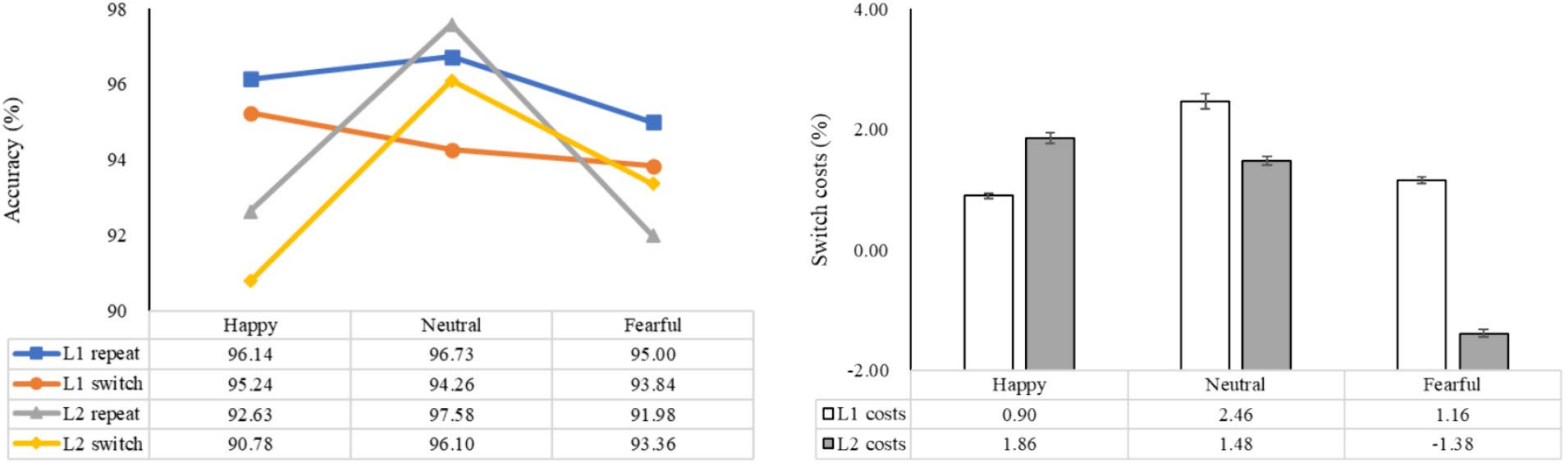
The mean accuracy of both L1 and L2 trials, as well as the switch costs (the difference in accuracy between switch trials and repeat trials) under different emotions. The left side displays the mean accuracy in L1 and L2 trials, while the right side demonstrates the magnitude of the costs for language switching in terms of accuracy.

The statistical analysis revealed significant main effects of emotion (*F* (2, 98) = 17.31, *p* < 0.001, *η_p_^2^* = 0.26), language (*F* (1, 49) = 13.42, *p* = 0.001, *η_p_^2^* = 0.22), and task (*F* (1, 49) = 6.62, *p* = 0.013, *η_p_^2^* = 0.12). There was a significant interaction between emotion and language (*F* (2, 98) = 13.61, *p* < 0.001, *η_p_^2^* = 0.22), while the interactions between emotion and task, as well as between language and task, were not statistically significant (*F* (2, 98) = 2.20, *p* > 0.05, *η_p_^2^* = 0.04; *F* (1, 49) = 1.09, *p* > 0.05, *η_p_^2^* = 0.02). The three-way interaction of emotion, language, and task was also not significant (*F* (2, 98) = 1.59, *p* = 0.210, *η_p_^2^* = 0.03), indicating that accuracy may not be a reliable indicator for evaluating how emotional priming influences language-switching performance. Further analysis showed that in L1 repetition trials, accuracy was significantly higher under the neutral condition compared to the fearful condition (*p* < 0.05). In L1 switch trials, accuracy did not differ significantly across the three emotional conditions (*ps* > 0.05). For both L2 repetition and switch trials, accuracy was significantly higher under the neutral condition than under the happy and fearful conditions (*ps* < 0.05). These results suggest that a fearful mood may negatively impact accuracy performance in L1 repetition, L2 repetition, and L2 switch, while a happy mood may negatively affect accuracy in L2 repetition and L2 switch (Figure 3).

A subsequent analysis examining the impact of emotion on language switching costs revealed non-significant main effects for both emotion and switch costs (*F* (2, 98) = 2.20, *p* > 0.05, *η_p_^2^* = 0.04; *F* (1, 49) = 1.09, *p* > 0.05, *η_p_^2^* = 0.02). The interaction between emotion and switch costs was not statistically significant (*F* (2, 98) = 1.59, *p* > 0.05, *η_p_^2^* = 0.03). Further analysis revealed a marginally significant difference between L1 and L2 switch costs under the fearful condition (*p* = 0.092), with L2 switch costs being marginally smaller than L1 switch costs, indicating a slight asymmetry between L1 and L2 switch costs under fearful emotion. There were no significant differences in L1 switch costs between the neutral, happy, and fearful conditions. However, for L2 switch costs, participants exhibited a marginally significant decrease compared to the neutral condition (*p* = 0.051). These findings suggest that accuracy may not be the primary indicator of bilingual switching costs under emotional priming, but rather that fear may potentially enhance L2 switching.

#### 3.1.2 Reaction times

**Figure 4:**
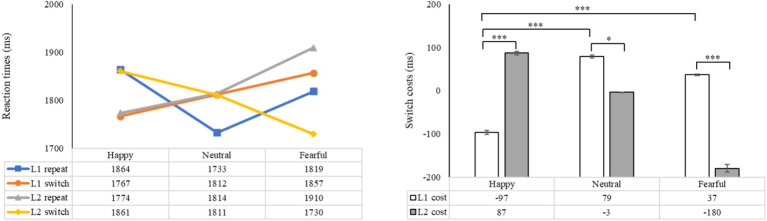
The mean reaction times and switch costs (the difference between switch and repeat trials in reaction times) under different conditions. The left side shows the mean reaction times in L1 and L2 trials, while the right side indicates the difference between switch trials and repeat trials in terms of reaction times. *p < 0.05, **p < 0.01, ***p < 0.001.

The statistical analysis revealed a significant main effect of emotion (*F* (2, 98) = 7.15, *p* = 0.001, *η_p_^2^* = 0.13), with non-significant main effects observed for language and task (*F* (1, 49) = 0.75, *p* = 0.391, *η_p_^2^* = 0.02; *F* (1, 49) = 2.56, *p* = 0.116, *η_p_^2^* = 0.05). Significant interactions were found between emotion and language (*F* (2, 98) = 3.93, *p* < 0.05, *η_p_^2^* = 0.07), emotion and task (*F* (2, 98) = 16.82, *p* < 0.001, *η_p_^2^* = 0.26), as well as between language and task (*F* (1, 49) = 4.21, *p* < 0.05, *η_p_^2^* = 0.08). The three-way interaction of emotion, language, and task was also significant (*F* (2, 98) = 46.10, *p* < 0.001, *η_p_^2^* = 0.49), indicating that reaction time serves as a reliable measure for assessing the influence of emotional priming on language-switching performance. Hypothesis 1 was confirmed. Subsequent analysis revealed that in L1 repetition trials, reaction times were shortest under the neutral condition, longest under the happy condition, and significantly different across the three emotional conditions (*ps* < 0.05). In L1 switch trials, reaction times were significantly shorter under the happy condition compared to the neutral (*p* < 0.05) and fearful (*p* < 0.001) conditions. In L2 repetition trials, reaction times under the happy and neutral conditions were significantly shorter than under the fearful condition (*ps* < 0.001). In L2 switch trials, reaction times were shortest under the fearful condition, longest under the happy condition, and significantly different across the three conditions (*ps* < 0.05). These findings suggest that, relative to the neutral condition, a happy mood enhanced L1 switching but negatively affected L1 repetition and L2 switching, while a fearful mood negatively impacted performance in L1 repetition and L2 repetition but positively influenced L2 switching (Figure 4).

A subsequent analysis examining the impact of emotion on language switching costs revealed significant main effects for both emotion and switch costs (*F* (2, 98) = 16.82, *p* < 0.001, *η_p_^2^* = 0.26; *F* (1, 49) = 4.21, *p* < 0.05, *η_p_^2^* = 0.08). The interaction between emotion and switch costs was also found to be significant (*F* (2, 98) = 46.10, *p* < 0.001, *η_p_^2^* = 0.49). Post-hoc analysis demonstrated significant differences in language switch costs between L1 and L2, depending on the emotional context. Specifically, under happy emotions, the switching cost was significantly lower for L1 compared to L2 (*p* < 0.001). Conversely, in neutral conditions, the switching cost for L1 was significantly higher than for L2 (*p* < 0.05), and under fearful emotions, the switching cost for L1 was also significantly higher than for L2 (*p* < 0.001). These findings indicate asymmetrical language switch costs between L1 and L2 across all emotional states. Compared to the neutral condition, switch costs for L1 were significantly reduced in the presence of happy emotions (*p* < 0.001). Additionally, switch costs for L1 under happy emotions were significantly lower than those under fearful emotions (*p* < 0.001). In contrast, compared to the neutral condition, the presence of happy emotions led to significantly higher switch costs for L2 (*p* < 0.01), while fearful emotions were associated with notably lower costs (*p* < 0.001). Moreover, the cost for L2 induced by happy emotions was significantly greater than that induced by fearful emotions (*p* < 0.001). The findings indicate that reaction time may be a crucial marker of bilingual switching costs influenced by emotional priming. Specifically, the results demonstrate that a positive emotional state enhances the switching process from L2 to L1 but hinders switching from L1 to L2. Conversely, a negative emotional state does not enhance switching to L1 but does aid in the processing of switching to L2.

### 3.2 ERP results

#### 3.2.1 P1 time window (75–105 ms)

**Figure 5:**
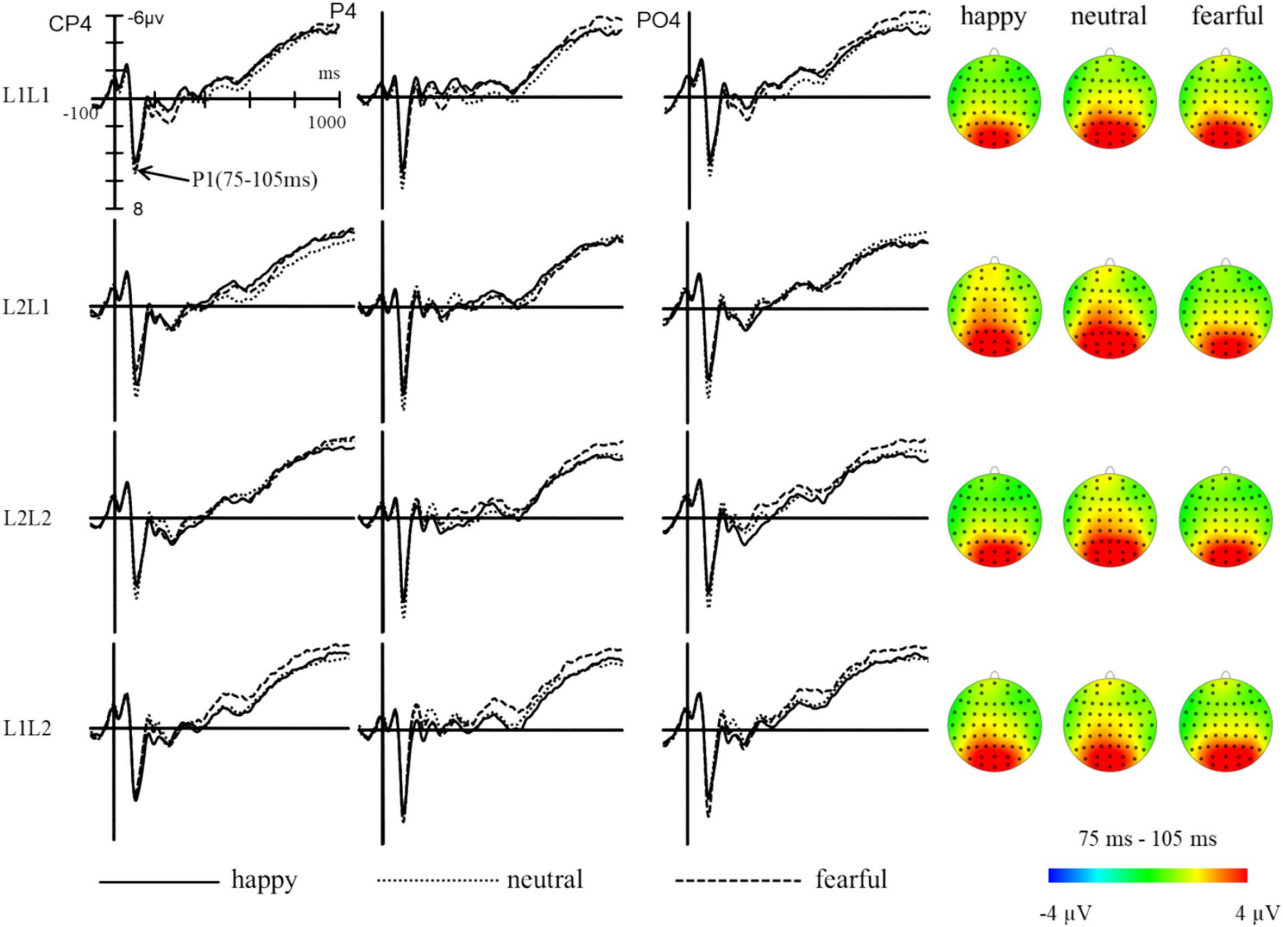
The grand average waveforms and topographic maps of L1 and L2 trials under different emotions in the P1 component (75–105 ms).

The statistical analysis indicated significant main effects of emotion (*F* (2, 98) = 23.10, *p* < 0.001, *η_p_^2^* = 0.32), task (*F* (1, 49) = 7.18, *p* < 0.05, *η_p_^2^* = 0.13), brain (*F* (2, 98) = 3.52, *p* < 0.05, *η_p_^2^* = 0.07), and sphere (*F* (2, 98) = 26.67, *p* < 0.001, *η_p_^2^* = 0.35), while the main effect of language was not significant (*F* (1, 49) = 1.11, *p* > 0.05, *η_p_^2^* = 0.02). The interaction between emotion and language (*F* (2, 98) = 1.85, *p* > 0.05, *η_p_^2^* = 0.04) was not significant, but the interactions between language and task (*F* (1, 49) = 4.91, *p* < 0.05, *η_p_^2^* = 0.09) and between emotion and task (*F* (2, 98) = 3.67, *p* < 0.05, *η_p_^2^* = 0.07) were significant. The three-way interaction of emotion, language, and task was significant (*F* (2, 98) = 8.88, *p* < 0.001, *η_p_^2^* = 0.15), indicating that the modulation effect of emotional priming on language-switching performance is prominently manifested during the P1 stage. Post-hoc analysis revealed that in L1 repetition trials, amplitudes were significantly larger under the neutral condition compared to the happy (*p* < 0.001) and fearful (*p* < 0.05) conditions, with no significant difference between the happy and fearful conditions. For L1 switch trials, amplitudes were significantly larger under the neutral condition compared to the happy (*p* < 0.01) and fearful (*p* < 0.001) conditions, while amplitudes were significantly higher in the happy condition compared to the fearful condition (*p* < 0.01). In L2 repetition trials, the amplitudes were significantly larger under the neutral condition compared to both the happy and fearful conditions (*ps* < 0.001), with no significant difference between the happy and fearful conditions. For L2 switch trials, there were no significant differences in amplitudes across the three emotional conditions (*ps* > 0.05). These results indicate that during the initial stage of visual attention allocation, a happy mood showed an advantage in L1 switch trials (Figure 5).

The five-way interaction of emotion, language, task, brain, and hemisphere was significant (*F* (8, 392) = 3.26, *p* = 0.001, *η_p_^2^* = 0.06). Further analysis revealed that when participants were primed with happy, neutral, or fearful emotions, there was a notable increase in L2 switch trials in certain areas of the brain’s hemispheres. Specifically, L2 switch trials in the parieto-occipital area of the left hemisphere, midline, or right hemisphere were all significantly larger than those in the parietal area (*ps* < 0.001), and the L2 switch trials in the parietal area of the left hemisphere, midline, or right hemisphere were all significantly larger than those in the central-parietal area (*ps* < 0.01). The findings indicate that the central-parietal, parietal, and parieto-occipital regions of the right hemisphere show increased sensitivity during the initial processing of language switching performance under the influence of emotional priming.

#### 3.2.2 N2 time window (200–300 ms)

**Figure 6:**
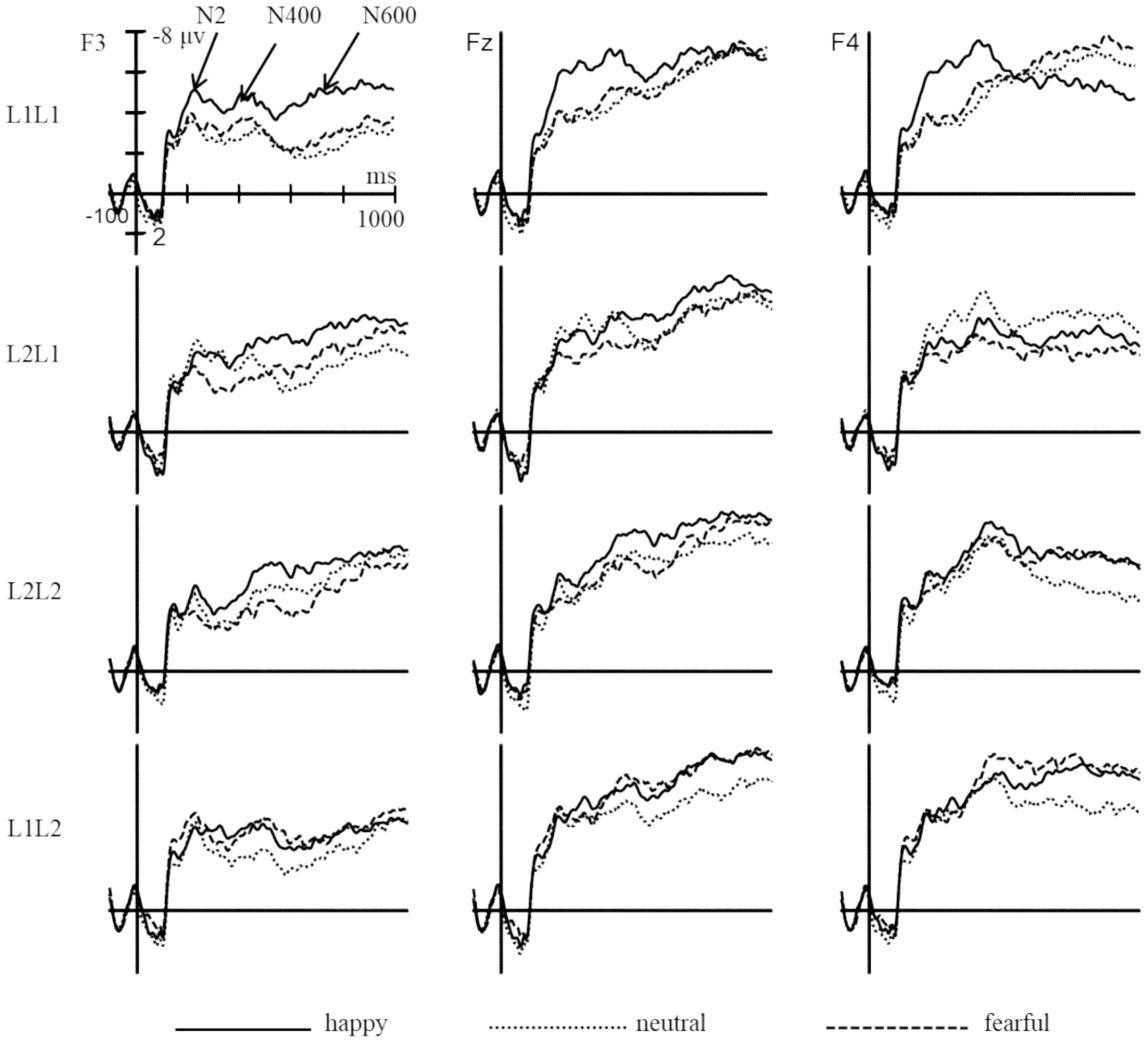
The grand average waveforms of L1 and L2 trials under different emotions in the N2 (200–300 ms), N400 (300–500 ms), and N600 (600–900 ms) components.

**Figure 7:**
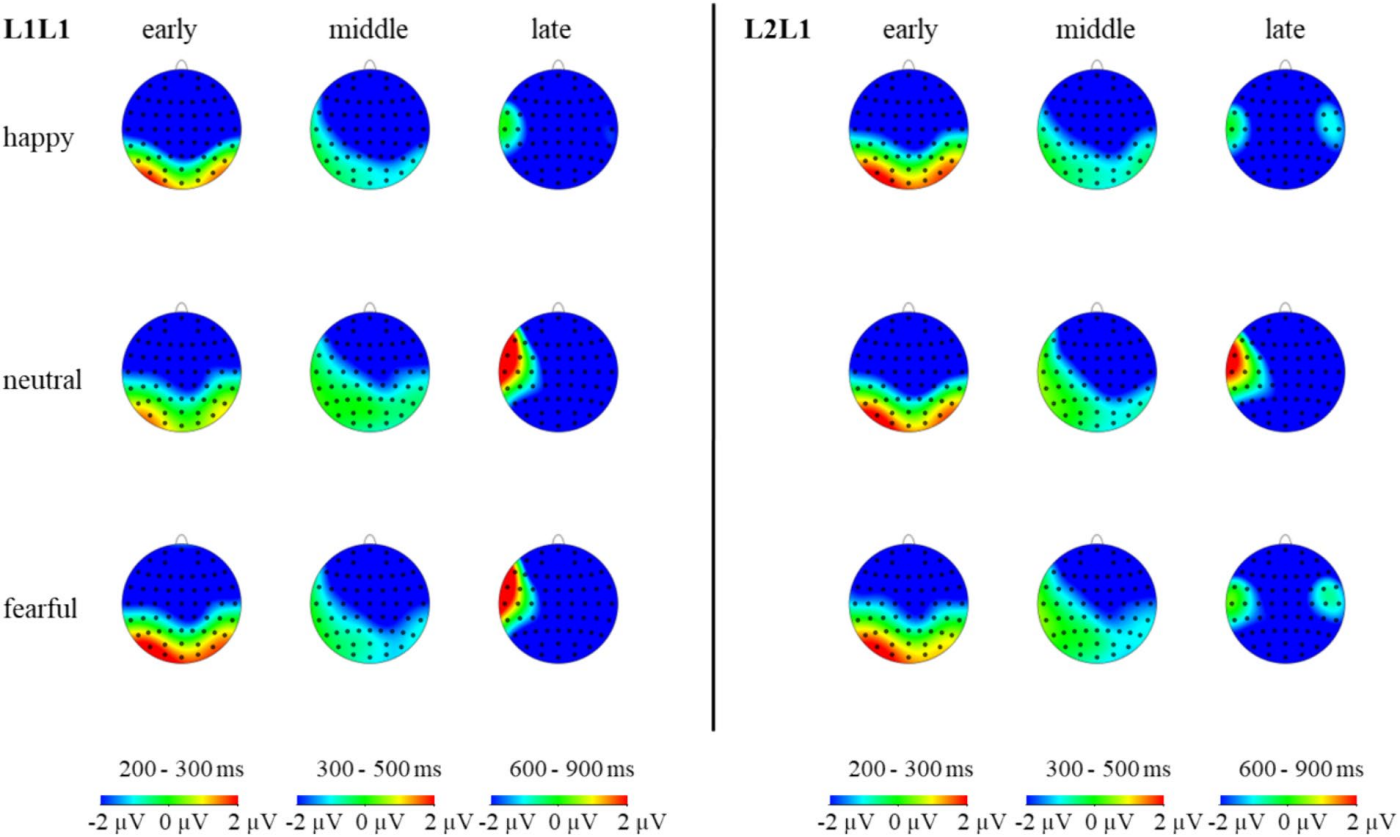
The topographic maps of L1 repeat and L1 switch trials under different emotions in the N2, N400, and N600 components.

**Figure 8:**
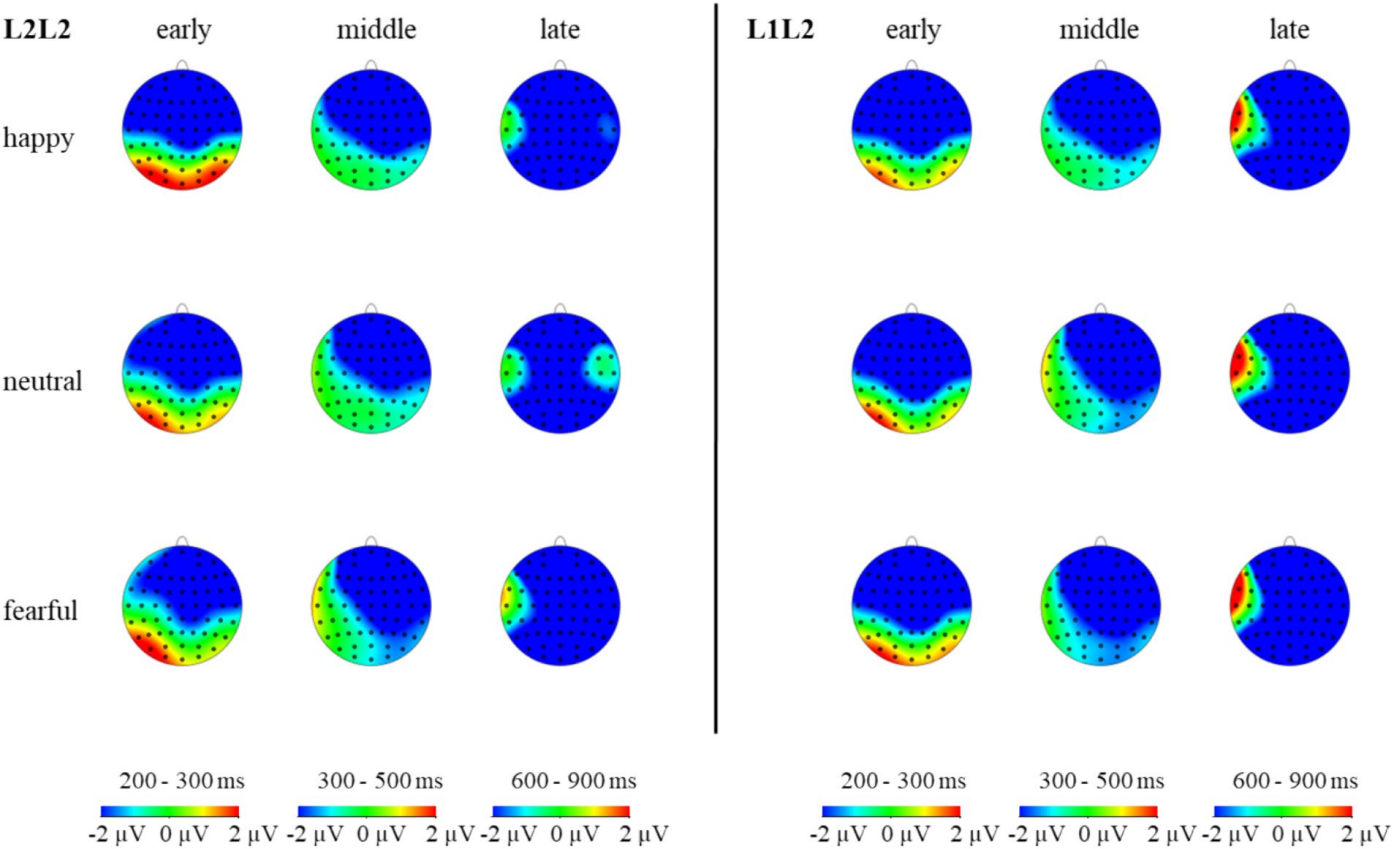
The topographic maps of L2 repeat and L2 switch trials under different emotions in the N2, N400, and N600 components.

The statistical analysis revealed significant main effects of emotion (*F* (2, 98) = 16.88, *p* < 0.001, *η_p_^2^* = 0.26), brain (*F* (2, 98) = 11.56, *p* < 0.001, *η_p_^2^* = 0.19), and sphere (*F* (2, 98) = 35.22, *p* < 0.001, *η_p_^2^* = 0.42) (Figures 6–8). However, the main effects of language (*F* (1, 49) = 0.95, *p* > 0.05, *η_p_^2^* = 0.02) and task (*F* (1, 49) = 2.38, *p* > 0.05, *η_p_^2^* = 0.05) were not significant. Significant interactions were found between emotion and language (*F* (2, 98) = 5.48, *p* < 0.01, *η_p_^2^* = 0.10), emotion and task (*F* (2, 98) = 7.82, *p* < 0.01, *η_p_^2^* = 0.14), as well as language and task (*F* (1, 49) = 21.90, *p* < 0.001, *η_p_^2^* = 0.31). The three-way interaction of emotion, language, and task was also significant (*F* (2, 98) = 7.41, *p* < 0.01, *η_p_^2^* = 0.13), indicating that the modulation effect of emotion priming on language-switching performance is evident during the N2 stage. Post-hoc analysis demonstrated that in L1 repetition trials, amplitudes were significantly larger under the happy condition compared to the neutral and fearful conditions (*ps* < 0.05), with no significant difference between the neutral and fearful conditions. In L1 switch trials, amplitudes were significantly larger under the happy (*p* < 0.01) and neutral (*p* < 0.001) conditions compared to the fearful condition, with no difference between the happy and neutral conditions. In L2 repetition trials, amplitudes were significantly larger under the happy condition compared to the fearful condition (*p* < 0.05). For L2 switch trials, no significant differences in amplitudes were observed across the three emotional conditions (*ps* > 0.05). These results imply that at the outset of attentional control and conflict processing, a state of happiness provides a benefit in L1 repetition, L1 switch, and L2 repetition.

The five-way interaction of emotion × language × task × brain × hemisphere did not reach significance (*F* (8, 392) = 0.84, *p* = 0.570, *η_p_^2^* = 0.02). This finding suggests that the influence of emotion on language switching was not evident in the brain and hemisphere during the N2 stage.

#### 3.2.3 N400 time window (300–500 ms)

The statistical analysis revealed significant main effects of emotion (*F* (2, 98) = 14.84, *p* < 0.001, *η_p_^2^* = 0.23), brain (*F* (2, 98) = 19.19, *p* < 0.001, *η_p_^2^* = 0.28), and sphere (*F* (2, 98) = 38.81, *p* < 0.001, *η_p_^2^* = 0.44). However, the main effects of language (*F* (1, 49) = 1.93, *p* > 0.05, *η_p_^2^* = 0.04) and task (*F* (1, 49) = 0.41, *p* > 0.05, *η_p_^2^* = 0.01) were not significant. Significant interactions were found between emotion and language (*F* (2, 98) = 3.24, *p* < 0.05, *η_p_^2^* = 0.06), emotion and task (*F* (2, 98) = 4.95, *p* < 0.01, *η_p_^2^* = 0.09), but not between language and task (*F* (1, 49) = 3.41, *p* > 0.05, *η_p_^2^* = 0.07). The three-way interaction of emotion, language, and task was significant (*F* (2, 98) = 5.56, *p* < 0.01, *η_p_^2^* = 0.10), indicating that the impact of emotion priming on language-switching performance is observable during the N400 stage. Post-hoc analysis revealed that in L1 repetition trials, amplitudes were significantly larger under the happy condition compared to the neutral (*p* < 0.001) and fearful conditions (*p* < 0.01), with no significant difference between the neutral and fearful conditions. In L1 switch trials, amplitudes were significantly larger under the happy and neutral conditions compared to the fearful condition (*ps* < 0.01), with no difference between the happy and neutral conditions. In L2 repetition trials, amplitudes were significantly larger under the happy condition compared to the fearful condition (*p* < 0.05). For L2 switch trials, amplitudes were significantly larger under the fearful condition compared to the neutral condition (*p* < 0.05). These findings suggest that during the earlier stage of lexical-semantic processing, a happy mood provides an advantage in the processing of L1 repetition and L2 repetition, while a fearful mood presents a disadvantage in L1 switch but an advantage in L2 switch. The findings also suggest that a positive mood aids in L1 repetition, L1 switch, and L2 repetition in the lexical-semantic processing stage. In contrast, a fearful mood boosts the L2 switch. These results are essential for investigating the cognitive mechanisms underlying bilingual language switching in comprehension, providing empirical evidence for the significant impact of fear emotions on lexical-semantic processing.

An analysis of the five-way interaction of emotion, language, task, brain, and hemisphere failed to reveal a significant result (*F* (8, 392) = 0.67, *p* = 0.721, *η_p_^2^* = 0.01). The finding indicates that the effect of emotion on language switching was not evident in the brain and hemisphere during the N400 stage.

#### 3.2.4 N600 time window (600–900 ms)

The statistical analysis indicated significant main effects of emotion (*F* (2, 98) = 7.23, *p* < 0.01, *η_p_^2^* = 0.13), brain (*F* (2, 98) = 25.47, *p* < 0.001, *η_p_^2^* = 0.34), and sphere (*F* (2, 98) = 46.54, *p* < 0.001, *η_p_^2^* = 0.49). However, the main effects of language (*F* (1, 49) = 0.34, *p* > 0.05, *η_p_^2^* = 0.01) and task (*F* (1, 49) = 1.18, *p* > 0.05, *η_p_^2^* = 0.02) were not statistically significant. Non-significant interactions were observed between emotion and language (*F* (2, 98) = 1.31, *p* > 0.05, *η_p_^2^* = 0.03), emotion and task (*F* (2, 98) = 0.04, *p* > 0.05, *η_p_^2^* = 0.001), as well as language and task (*F* (1, 49) = 1.17, *p* > 0.05, *η_p_^2^* = 0.02). The three-way interaction of emotion, language, and task was also not significant (*F* (2, 98) = 1.84, *p* > 0.05, *η_p_^2^* = 0.04), indicating that the impact of emotion priming on language-switching performance is not notable during the N400 stage. The modulation of bilingual switching by emotion priming exhibits diverse characteristics across different time windows, as evidenced by the results of the P1, N2, N400, and N600. Therefore, Hypothesis 2 has been confirmed. Post-hoc analysis revealed that in L1 repetition and L1 switch trials, amplitudes did not differ across the three emotional conditions (*ps* > 0.05). In L2 repetition trials, amplitudes were significantly larger under the happy condition compared to the neutral condition (*p* < 0.05). For L2 switch trials, amplitudes were significantly larger under the fearful condition compared to the neutral condition (*p* < 0.01). These results suggest that during the later stage of deeper levels of semantic processing, a happy mood provides an advantage in the processing of L2 repetition, while a fearful mood offers a clear advantage in L2 switch. This finding is significant as it provides evidence that, in the higher levels of the semantic processing stage, a fearful mood still facilitates bilingual language comprehension processes. This is a novel contribution to the impact of emotion on bilingual language switching.

A five-way interaction of emotion × language × task × brain × hemisphere did not reach significance (*F* (8, 392) = 1.68, *p* = 0.101, *η_p_^2^* = 0.03). The finding suggests that the presence of emotion did not significantly affect language switching in the brain and hemisphere during the N600 stage.

#### 3.2.5 The comparison of bilingual switch costs for different emotions at N2, N400, and N600

**Figure 9:**
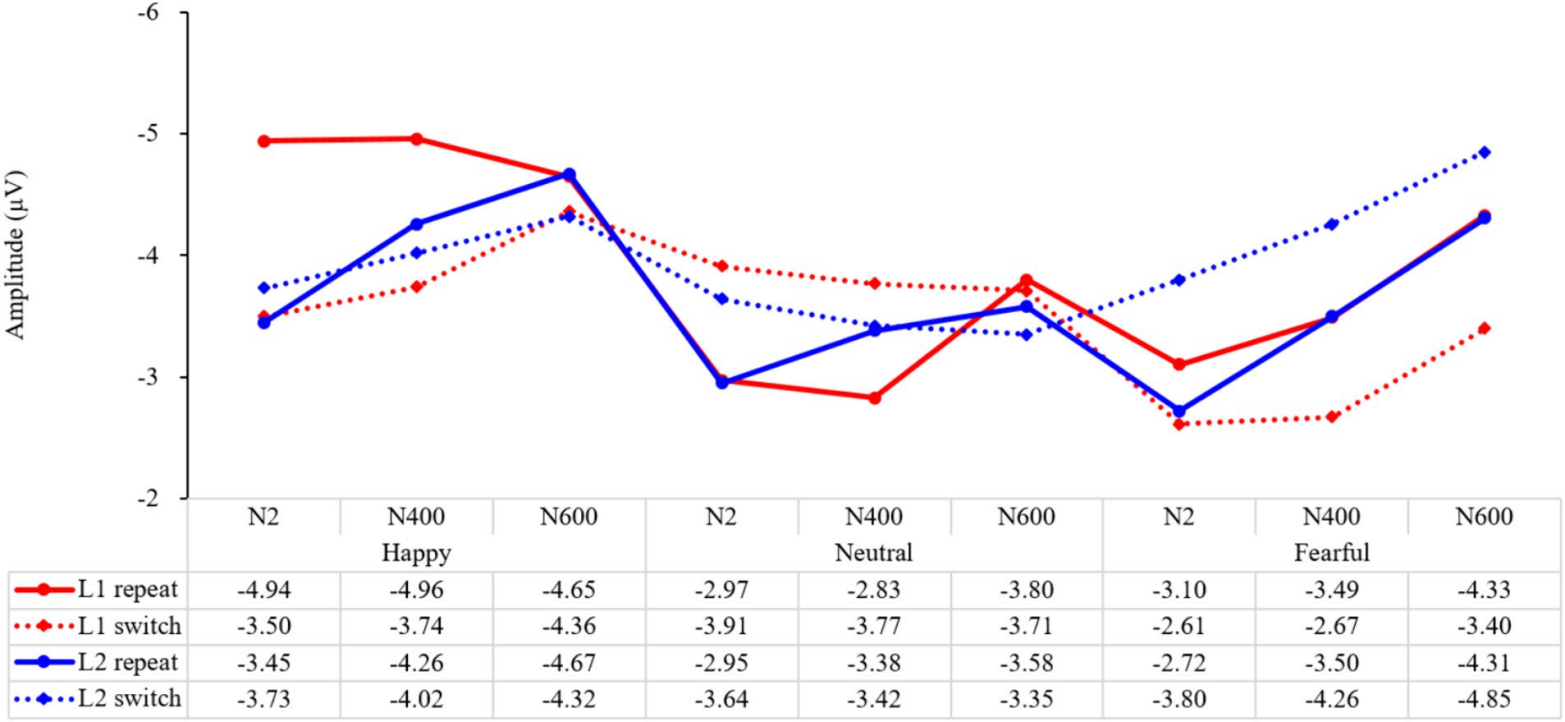
The mean amplitude of different trial types at different stages under different emotions.

Since the P1 stage is mainly influenced by the physical properties of the emotional stimulus and is considered part of the exogenous component, while the N2, N400, and N600 stages are linked to endogenous components in traditional research and may better reflect the process of inhibitory control, we performed a repeated-measures ANOVA on stage (N2, N400, N600) and cost (L1 switch costs, L2 switch costs) with happy, neutral, or fearful emotions, respectively. The results revealed, under a happy mood, a significant main effect of cost (*F* (1, 147) = 7.07, *p* = 0.009, *η_p_^2^* = 0.05) (Figure 9). The interaction of stage and cost was marginally significant (*F* (2, 147) = 2.44, *p* = 0.090, *η_p_^2^* = 0.03). Further analysis revealed that L1 switch costs were significantly higher at N2 and N400 than at N600 (*p* = 0.014, *p* = 0.046), suggesting that happy emotion modulated inhibition, releasing more in the earlier stages. In contrast, L2 switch costs did not differ among N2, N400, and N600 (*ps* > 0.05), indicating that happy emotion did not lead to a significant difference in applying inhibition across different stages.

An ANOVA analysis of stage and cost revealed a significant main effect of stage (*F* (2, 147) = 3.92, *p* = 0.022, *η_p_^2^* = 0.05) when participants were in a neutral emotional state. However, the interaction of stage and cost was not significant (*F* (2, 147) = 0.65, *p* = 0.523, *η_p_^2^* = 0.01). Further analysis showed that the switch costs for L1 at the N2 and N400 stages were marginally higher than those at N600 (*p* = 0.051, *p* = 0.050), suggesting that inhibition releasing was more prominent in the earlier stages of the language task schema competition and lexical selection response. Additionally, the switch costs for L2 at N2 were marginally larger than those at N600 (*p* = 0.061), indicating that in a neutral state, inhibition application was more evident in the language task schema phase than in the deep semantic processing phase.

Under a fearful mood, the results showed a significant main effect of cost (*F* (1, 147) = 23.43, *p* < 0.001, *η_p_^2^* = 0.14), indicating that fear had a noticeable impact on language switching. However, the interaction of stage and cost was not significant (*F* (2, 147) = 0.01, *p* = 0.988, *η_p_^2^* = 0.001), suggesting that the effect of fear on language switching remained consistent across different processing stages. Further analysis revealed that there was no difference in switch costs for the first language among N2, N400, and N600 (*ps* > 0.05), indicating that inhibition release remained constant throughout the language-switching process. Similarly, there was no difference in switch costs for the second language (*ps* > 0.05), demonstrating that inhibition application was persistent throughout the language-switching process. It is worth noting that the switch costs for the second language were consistently higher than those for the first language at all stages. This suggests that fear had a lasting effect on language switching, making it helpful for activating inhibition but harmful for suppressing it, regardless of the processing stage.

In summary, the impact of emotions on language-switching performance is diverse. Positive emotions have a greater influence on releasing inhibition in the task schema competition and lexical selection response phases, while negative emotions have a persistent effect throughout the entire language-switching process. In essence, positive emotions consistently modulate inhibition across all stages, but they lead to a significant increase in inhibition release during the language task schema and lexical selection phases. On the other hand, negative emotions are characterized by consistent inhibition and release throughout the language-switching process, with a greater emphasis on applying inhibition rather than releasing it.

### 3.3 Correlations

In this study, our specific focus was on switch trials to explore inhibition in the context of language switching. Considering the limited sensitivity of accuracy as a measure, we chose to analyze reaction times (RTs) instead. The results of the correlation analyses are presented in Table 2. When participants were in a happy mood, the correlation between reaction times and the amplitudes of L1 switch trials was significant at both the N2 (*r* = 0.38, *p* = 0.007) and N400 (*r* = 0.36, *p* = 0.010) stages. The correlation between reaction times and the amplitudes of L2 switch trials was significant at the P1 (*r* = −0.37, *p* = 0.008), N2 (*r* = 0.32, *p* = 0.022), and N400 (*r* = 0.29, *p* = 0.042) stages. These results imply that, under positive emotion, participants in the N2 and N400 stages may allocate more cognitive resources and effort to accessing their previously suppressed first language. Meanwhile, participants in the P1, N2, and N400 stages may utilize more attentional and cognitive resources to resist strong interference from their first language. When participants were in a neutral mood, the correlation between reaction times and the amplitudes of L1 switch trials was significant at the N2 stage (*r* = 0.31, *p* = 0.028). For L2 switch trials, the correlation was marginally significant at both the N2 (*r* = 0.28, *p* = 0.052) and N400 (*r* = 0.27, *p* = 0.059) stages. These findings suggest that, under neutral conditions, the N2 stage requires participants to invest more cognitive effort in releasing the previously suppressed first language, while the N2 and N400 stages require participants to spend more effort suppressing interference from their mother tongue. When participants were exposed to a fearful mood, the correlation between reaction times and the amplitudes of L1 switch trials was significant at the P1 stage (*r* = −0.31, *p* = 0.027). This finding suggests that shorter reaction times during early visual sensory processing are associated with higher P1 amplitudes, indicating an advantage in negative emotional processing for L1 switching. For L2 switch trials, the correlation was marginally significant at the P1 stage (*r* = −0.26, *p* = 0.073), significant at the N2 stage (*r* = 0.33, *p* = 0.018), and marginally significant at the N400 stage (*r* = 0.25, *p* = 0.083). These results suggest that when experiencing fear, individuals must exert cognitive resources to retrieve their previously suppressed native language during the P1 and N400 phases. In contrast, during the P1, N2, and N400 phases, greater neural and cognitive resources are required to inhibit interference from the mother tongue. Nevertheless, the complexity of language switching indicates that ERP data alone is insufficient to account for reaction times completely. These times encompass more than just the activation and suppression of inhibition but also involve other cognitive processes.

**Table 2:** A summary of Pearson correlations between behavioral measures (RT, reaction times) and ERP measures (amplitudes) for L1 switch (L1S) and L2 switch (L2S) trials under different conditions (*N* = 50).

Emotions		P1-L1S	N2-L1S	N400-L1S	N600-L1S		P1-L2S	N2-L2S	N400-L2S	N600-L2S
Happy	L1S-RT	–	**	**		L2S-RT	–**	*	*	
Neutral	L1S-RT		*	–		L2S-RT			–	–
Fearful	L1S-RT	–*		–		L2S-RT	–	*	–	

**<0.01; *<0.05; –, marginal significance.

## 4 Discussion

This research utilized event-related potentials (ERPs) to investigate the influence of emotion priming on language switching at different processing stages. The results from behavioral data revealed that a positive mood enhanced the accuracy and speed of switching to the first language, while a fearful mood improved the efficiency of switching to the second language. The electrophysiological data showed that emotion priming had a notable impact on the P1, N2, N400, and N600 stages. Positive emotions were found to benefit L1 switching during the early stages of visual attention allocation, conflict processing, and lexical-semantic processing. In contrast, negative emotions showed a greater advantage for L2 switching in lexical-semantic processing and deeper levels of semantic processing. These findings will be further discussed in the subsequent sections.

### 4.1 Emotion priming could modulate language switch costs through moderating inhibitory control

The inhibitory control model proposed by Green (1998) highlights the significant role of inhibitory control in bilingual language switching. This study investigated the language-switching performance of Chinese-English bilinguals after priming them with happy, neutral, and fearful emotions. The results revealed distinct asymmetrical switch costs for the happy and fearful conditions. Specifically, a happy mood led to larger L2 switch costs but smaller L1 costs, while a fearful mood resulted in smaller L2 switch costs but larger L1 switch costs. These findings indicate that distinct emotions may influence inhibitory control in their unique ways, resulting in distinct patterns of language switching costs.

Mention should be made of the fact that the switching cost is generally recognized as a measure of switching ability, with lower costs indicating increased flexibility in task switching (Slama et al., 2015; Schwenke et al., 2020; Carvalho et al., 2022). This study evaluates participants’ switching ability by examining switch costs, focusing on accuracy and reaction time differences between switch and repetition trials. A lower switch cost reflects enhanced switching ability, whereas a higher cost suggests limitations in this aspect. The findings of this study align with previous research indicating that different emotions can affect inhibitory control and subsequent task switching (Bialystok, 2017; Liu et al., 2021; Wang et al., 2023). For example, Liu et al. (2021) experimented to investigate the impact of different emotional states (anger, fear, pleasure, and neutral) on performance in switching mathematical strategies. Their results revealed that participants responded most rapidly when experiencing pleasure, while those in a state of fear exhibited the slowest reaction times. This suggests that positive emotions may have a favorable influence on inhibitory control and task switching. However, our study uncovered that while positive emotions provided a benefit for L1 switching, they posed a challenge for L2 switching. Conversely, fearful emotions impeded L1 switching but enhanced L2 switching. This research sheds light on the complex impact of positive and negative emotions on inhibitory control, expanding current knowledge on task switching and introducing a unique insight from our investigation.

The current study investigated how emotion priming affects inhibitory control in language switching. It was discovered that happy and fearful moods resulted in different unbalanced patterns between L2 and L1 switch costs, indicating that different emotions may have their unique modulating mechanisms in language switching performance. These findings again support the notion that emotions can influence language switch costs by moderating inhibitory control.

### 4.2 The modulation of positive emotions occurs from the early stage of visual attention to the later stage of semantic processing

According to the IC model proposed by Green (1998), increased inhibition is engaged when transitioning to L2, resulting in a prolonged switching time back to L1. This suggests inhibitory control may modulate inhibition-related language switching variables, indicating an interplay among these factors in the P1, N2, N400, and N600 data. In the current study, significant interactions were observed among emotion, language, and task in the P1, N2, and N400 periods. Specifically, a happy mood was associated with an increased P1 amplitude during L1 switching, larger N2 amplitudes during L1 repetition, L1 switching, and L2 repetition, as well as greater N400 amplitudes during L1 repetition, L1 switching, and L2 repetition, along with a larger N600 during L2 repetition. Positive emotions were beneficial for L1 switching, primarily in the initial stages of visual attention, conflict processing, and lexical-semantic processing. However, this advantage was not observed in the later stages of deep levels of semantic processing. In conclusion, happy emotions were advantageous for completing the repetition task in both L1 and L2, as well as for releasing previously suppressed inhibition. The beneficial effect of happy emotions is most prominent from the early stages of visual attention allocation to the lexical-semantic processing stages. This effect diminishes in the later stages of higher semantic processing. The advantage of L1 switching facilitated by a happy mood may be attributed to a sense of security. Prior research has demonstrated that positive emotions can boost feelings of security, resulting in heightened attention to the current context and well-known tasks (Bless et al., 1996; Clore and Palmer, 2009; Vesa et al., 2017). Therefore, regulating positive emotions may have a beneficial impact on L1 switching, facilitating the release of the dominant language that was previously suppressed.

The results of the current study indicate that priming with positive emotions can modulate switch costs during the initial stage of visual attention allocation, conflict resolution, lexical selection response, and subsequent semantic processing, consistent with prior research (Guo et al., 2013a, 2013b). Guo et al. (2013a, 2013b) observed that in trilingual individuals, the *n*-2 repetition trials elicited a more negative ERP component than the *n*-2 non-repetition trials in the early stage of the lexical selection response phase. Based on previous evidence and our study findings, it is hypothesized that the primary advantage of experiencing happiness lies in its capacity to release previously suppressed inhibitions rather than in its ability to inhibit interference from the dominant task. Furthermore, the beneficial effect of happiness in releasing inhibitions may not be present during higher levels of semantic processing.

### 4.3 Negative emotion priming demonstrates sustainable effects from the initial visual attention processing to the subsequent deeper levels of semantic processing

What processing mechanisms are reflected by the P1 during the initial stage of the language-switching process? In the current study, the P1 amplitude was observed to be influenced by language and task when primed with a fearful mood. It is speculated that visual attention may have a significant impact at the onset of language switching, and fearful emotion exhibits a bias toward a negative processing advantage. Previous research has shown that the P1 plays a role in visual attention and early emotional stimulus processing, which is essential for visual sensory processing. It is typically localized in the parietal-occipital regions and is associated with an individual’s arousal state (Luck et al., 2000). When individuals are exposed to negative emotional stimuli, the amplitude of the P1 typically increases (Mueller et al., 2009). The current study uncovers a significant interaction of the P1 with language and task, indicating that participants exhibit heightened visual perception of negative information subsequently influencing their language-switching performance.

The significant interplay among emotion, language, and task during the N2 stage suggests the involvement of the N2 component in attentional control in language switching, as evidenced in prior studies (Verhoef et al., 2009). This reaffirms its role in detecting competition between language task schemas, a notion supported by various tasks such as the go/no-go task, the Stroop task, the flanker task, and the Simon task, highlighting the N2’s pivotal role in monitoring conflict during attentional processes. The N400 is a cognitive potential utilized to investigate language processing in the brain, particularly its sensitivity to semantic violations and its reflection of language comprehension and processing abilities (Hagoort and Brown, 2000). Magne et al. (2016) argue that a violation of speech rhythm can also evoke the N400 in the central-frontal region, a language-specific negative wave associated with speech rhythm expectations (Zhang and Zhang, 2019). Irregular rhythm patterns may exacerbate difficulties in semantic access and integration (Henrich et al., 2013). In essence, the amplitude of the N400 increases when language systems are switched, indicating heightened language processing challenges, with its magnitude reflecting the consistency between language sequences and expectations. Based on the literature and our study, it is plausible to suggest that inhibitory control may play a role in differentiating the correct word from distractor words during participants’ engagement in a word-picture matching task. As for the N600, a negative wave, it is reported to typically be triggered at 600–800 ms after encountering incongruent semantic structures (Chan et al., 2012). Shibata et al. (2009) proposed that this component resembles the N400 and could be interpreted as a subsequent N400 response in the later stage, associated with more intricate semantic processing and comprehension. The impact of fearful emotions on the N2, N400, and N600 stages in language switching, specifically in L2 switch trials, was the focus of this study. Our study’s findings indicate that fearful emotions may impact language-switching performance at these stages, as there is a notable interaction between language and task that continues during language-switching due to the ongoing conflict between L1 and L2 task types.

The present study found a significant correlation between the amplitude of L2 trials and the reaction time of L2 trials when exposed to fearful emotion priming across various stages (P1, N2, N400, and N600). A higher amplitude is linked to a quicker response time, suggesting that negative emotions have a positive impact on processing new information. The profound impact of negative emotions, particularly fear, is prominently evident during the N400 and N600 stages, emphasizing their role in seeking safety and avoiding threats. Fear, a fundamental aspect of human experience, plays a crucial role in pursuing benefits, mitigating risks, and anticipating dangers, with significant biological and evolutionary implications (Gable et al., 2022). This study revealed the advantageous impact of fearful emotions, especially on L2 switch trials, demonstrating their robust capacity to suppress interference from the dominant language during encounters with dangerous circumstances. However, the bottom-up processing nature of fearful emotions led individuals to excessively focus on dealing with novel stimuli in L2 switching tasks, depleting cognitive resources and impeding timely disengagement from the current task. Consequently, individuals lacked the flexibility required to complete the entire switching task, leaving insufficient cognitive resources to lift the previously suppressed inhibition when transitioning back to the native language. In the future, it will be crucial to investigate how people can effectively manage their attentional resources and cognitive effort in applying and releasing inhibition. Additionally, it is important to understand how to shift the unidimensional advantage of negative emotions in applying inhibition into a bidimensional advantage of applying and releasing information.

### 4.4 Theoretical implications

The prevailing IC model posits that inhibition is responsible for switch costs. We aim to augment this model from a novel perspective. The IC model suggests that inhibition not only plays a crucial role in language switching, but also that inhibitory control is adaptable and modifiable. Our findings support this notion, demonstrating that emotions influence inhibition and language-switching proficiency. Specifically, our study observed that positive emotions were beneficial for L1 switching in the early stages of visual attention allocation, conflict processing, and lexical-semantic processing. In contrast, negative emotions exhibited a greater advantage for L2 switching in lexical-semantic processing and deeper levels of semantic processing. This indicates that emotions can modulate inhibition to enhance language-switching proficiency, particularly when switching to a less dominant language.

The IC model (Green, 1998) is based on the supervisory attention system (SAS) model, which posits that human behavior is regulated by contention scheduling and supervisory attention involving inhibition (Norman and Shallice, 1986). Contention scheduling controls prevent conflicting schemata from vying for the same cognitive resource through inhibitory mechanisms (Shallice and Burgess, 1991). Meanwhile, supervisory attentional control, a high-level conflict resolution system, inhibits non-relevant stimuli from focusing on the current task. Task-switching research has shown that the SAS is crucial in inhibiting irrelevant targets (Mueller, 2013; Grange and Houghton, 2014). In language switching, contention scheduling and supervisory attentional control are akin to language task schema competition and lexical-semantic processing stages. Hence, emotions can influence inhibitory control and language switching performance, making language switch costs flexible and modifiable by different emotions.

## 5 Conclusion

This study investigated how emotions can influence language switching between languages. The findings revealed that emotions can impact language switching by modulating inhibitory control. Positive emotions facilitated the release of inhibition for L1 switching during the early stages of visual attention allocation, conflict processing, and lexical-semantic processing. In contrast, negative emotions exhibited a significant advantage in recruiting inhibition for L2 switching during lexical-semantic processing and more complex levels of semantic processing. This study is the first to offer significant electrophysiological evidence of how emotional priming can affect the effectiveness of language switching.

## Data Availability

The original contributions presented in the study are included in the article/supplementary material, further inquiries can be directed to the corresponding author.

## Footnotes

^1^See http://crl.ucsd.edu/experiments/ipnp.

^2^ http://www.macbrain.org
